# P-826. Impact of Antimicrobial Stewardship Prospective Audit on the Treatment of Staphylococcus aureus Bloodstream Infections

**DOI:** 10.1093/ofid/ofae631.1018

**Published:** 2025-01-29

**Authors:** Eunice Kim, Ryan W Chapin, Christopher McCoy

**Affiliations:** Community Health Network, Indianapolis, Indiana; Beth Israel Deaconess Medical Center, Boston, MA; Beth Israel Deaconess Medical Center, Boston, MA

## Abstract

**Background:**

Staphylococcus aureus (SA) is a serious cause of bloodstream infection (BSI) associated with significant morbidity and mortality. Empiric treatment of Gram-positive cocci (GPC) BSIs varies across institutions. Vancomycin, compared to beta-lactams for the treatment of MSSA, is associated with longer durations of bacteremia and higher relapse rates. At our center, a lack of rapid phenotypic diagnostics technology (RPDT) limits prompt optimization. A pre- and post- implementation analysis was performed to assess the impact of a guideline driven stewardship pharmacist intervention on GPC BSI treatment.

**Figure:**
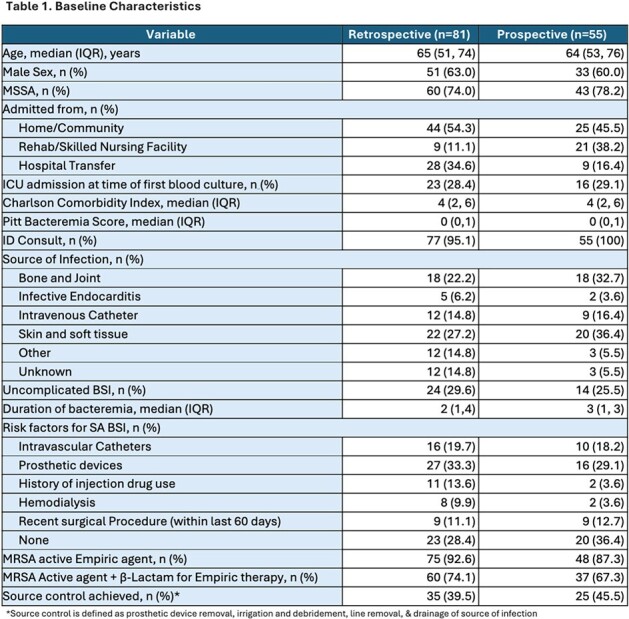

**Methods:**

Patients with GPCs in blood cultures were identified using a third-party pharmacovigilance platform. Patients were included for if they grew SA in at least 1 blood culture bottle and excluded if they had polymicrobial infections or if they were placed on comfort measures before antimicrobial therapy was tailored. The primary endpoint was time to optimal therapy, defined as the narrowest effective therapy. Secondary endpoints included time to Infectious Diseases (ID) consultation after GPC identification, duration of BSI, and rate of stepdown to oral antimicrobials for patients with uncomplicated BSI.

**Figure:**
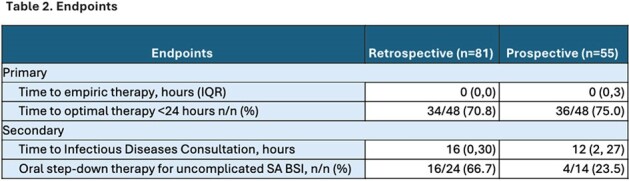

**Results:**

From June 2023 to April 2024, 136 patients were identified. Eighty-one patients were included in the pre-guideline group (before December of 2023), and 55 patients were included in the prospective review. Baseline demographics were similar between the two groups (table 1). Patients receiving optimal therapy within 24 hours was 70.9% in the pre-guideline group and 75% in the post-guideline group. Step-down to oral therapy occurred in 66.7% and 23.5% patients, respectively.

**Conclusion:**

We found implementation of a SA BSI guideline with steward follow up did not significantly alter time to empiric therapy, ID consults, or number of patients on tailored therapy within 24h of susceptibilities. Since most patients in our analysis received an infectious diseases consult, fewer opportunities for independent stewardship intervention existed. RPTD may play a potential role to decrease time to optimal therapy at our institution, results will be shared to support the onboarding of this stewardship tool.

**Disclosures:**

**All Authors**: No reported disclosures

